# Optimizing public health management with predictive analytics: leveraging the power of random forest

**DOI:** 10.3389/fdata.2025.1574683

**Published:** 2025-07-10

**Authors:** Hongman Wang, Yifan Song, Hua Bi

**Affiliations:** ^1^School of Humanities, Southeast University, Nanjing, China; ^2^Faculty of Humanities and Social Sciences, Macao Polytechnic University, Macau, Macao SAR, China; ^3^General Hospital of the Central Theater of the People's Liberation Army, Wuhan, China

**Keywords:** predictive analytics, random forest, public health, health risk factors, machine learning, community-based health management, older adults

## Abstract

Community health outcomes significantly impact older populations' wellbeing and quality of life. Traditional analytical methods often struggle to accurately predict health risks at the community level due to their inability to capture complex, non-linear relationships among various health determinants. This study employs a Random Forest Algorithm (RFA) to address this limitation and enhance the predictive modeling of community health outcomes. By leveraging ensemble learning techniques and multi-factor analysis, this study aims to identify and quantify the relative contributions of key health indicators to risk assessment. The study begins with comprehensive data collection from diverse health sources, followed by a systematic preprocessing stage, which includes resolving missing values, normalizing variables, and encoding categorical features. Using bootstrap sampling, multiple decision trees were trained on random subsets of health data, ensuring variability in the model learning. The trees grow to full depth and aggregate their predictions to enhance the accuracy. An out-of-bag (OOB) error estimation was applied to refine the model and provide unbiased performance assessments, ensuring robust generalization to unseen data. The proposed model effectively analyzes key health indicators, ranking the feature importance to determine the most influential predictors of health risks. Results indicate that RFA achieves an accuracy rate of 92%, outperforming conventional prediction methods in terms of precision and recall. These findings underscore the efficacy of Random Forest in identifying critical health risk factors, paving the way for targeted and data-driven public health management strategies and interventions tailored to older adults.

## 1 Introduction

Community health is an important aspect of public health. It identifies issues related to the wellbeing of the population within a specific geographical area and identifies different strategies for mitigating risks. Timely and early detection of these problems, especially in terms of health, can reduce the likelihood of epidemics and provide better planning in the event of a need or disaster (Pazzaglia et al., 2023). The ability of community health systems to anticipate and manage community health risks at an early stage is crucial. This is because early recognition can prevent disease, reduce healthcare costs, and improve the overall quality of life (Wulandari et al., 2023). However, predicting a community's health outcomes is a complex task due to the large number of disparate datasets consisting of demographic information, lifestyle factors, and environmental conditions. Due to rapid changes in the socio-economic and health status of the population, the amount of health data is increasing every day. Due to their diversity and unevenness, manual methods cannot process these data. Advancements in machine learning models (MLMs) provide powerful tools to analyze this data (Amiri et al., 2023) and generate meaningful reports. This study leverages the capabilities of RFA to improve community health outcomes and uncovers advanced predictive models that can lead to better health management strategies (Suresh and Herzog, 2024). Traditional methods for predicting community health outcomes are based on different models, including linear regression (LR), logistic regression (LogR), and decision trees (DT) (Joubert and Reid, 2023). These methods are used to analyze public health data but have significant limitations in terms of data quality and characteristics. The first is the LR approach, which uses linear relationships between variables to process data, but its problem is that it oversimplifies complex interactions in health data. The second method is LogR, which is useful for binary outcomes but difficult for multi-class predictions. The third is DT, which is flexible and has an increased ability to process data but can lead to overfitting problems and performing poorly on new and unseen data (Upadhyay et al., 2023). These models also face challenges when working with high-dimensional data and become less effective as the number of variables increases. Overfitting is another major problem that complicates the model and makes it impossible to generalize to new data. In addition, traditional models require manual feature selection, which is a time-consuming process that often misses important interactions between variables (An et al., 2023).

To address these challenges, MLM offers promising solutions such as Decision Tree (DT), Support Vector Machine (SVM), and RFA. This is an ensemble learning technique that builds multiple decision trees from a single node. It is constructed in such a way that each tree is designed using a random subset of data with a selected number of features (Vellela et al., 2023). This approach enables the capture of complex non-linear relationships between variables, thereby reducing the risk of overfitting and efficiently handling high-dimensional data. Unlike conventional models, RFA does not require manual feature selection, and automatically identifies the most important features. It also provides information on how important the feature is in generating insights. The system makes the model more robust and performs well on both trained and unseen data using multiple trees to interpret the model's predictions (Vieira et al., 2022).

This study analyzed a large and comprehensive health dataset using RFA. The data in the dataset is collected from various health-related sources such as hospitals and communities. It includes a variety of health indicators such as demographic information, lifestyle factors, and medical history. The initial dataset consists of errors and anomalies, which are removed using data cleansing and preprocessing techniques and ensuring that the data is clean and ready for analysis. This step involves dealing with missing values, normalizing variables, and coding categorical features. After preprocessing, the data is divided into training and test sets. The training dataset was trained using the proposed RFA model, and the hyperparameters were fine-tuned using cross validation. Once trained, the model is tested on an invisible test set and predictions are made using both risky and risk-free classes. The objectives of this study are as follows:

- Demonstrate the application of RFA in predicting community health outcomes by analyzing complex and high-dimensional health datasets. It also highlights how RFA can be effectively used to capture non-linear relationships between health indicators.
- Identify key health indicators that influence community health outcomes and determine feature importance through the RFA. It provides insights into which variables are most crucial for predicting health risks, which aids in targeted public health interventions.
- It provides a comprehensive comparison between RFA and traditional predictive models such as linear regression, logistic regression, and decision trees. This shows that RFA outperforms these traditional models, particularly in handling multi-class predictions and reducing the risk of overfitting.

The rest of this study is structured as follows. The second part of the next section provides a detailed literature review and discusses the current state of predictive analytics in public health. Section 3 describes the data, preprocessing steps, and architecture of the proposed RFA. It also explains the model's training and validation processes. Section 4 explains the results of the study. It includes a comparison of random forests with other predictive models. The study summarizes recommendations for future research and application of public health policy in Section 5.

## 2 Literature review

The literature on predictive analytics in public health is extensive and accommodates various machine-learning techniques that have been applied to improve health outcome predictions. This section reviews the key studies used in the forecasting of community health.

### 2.1 Traditional statistical methods

A study conducted by Zhou et al. (2023) evaluates the application of logistic regression (LogR) in predicting the incidence of diabetes within a community. This method uses LogR to model the probability of diabetes occurrence and considers it as a function of several predictors, such as age, BMI, and family history. This shows the capability of LogR to classify individuals as high- or low-risk. Apart from the benefits of the utilized scheme, it highlighted the limitations of LogR in handling multi-class predictions and non-linear relationships among variables. The author pointed out that LogR assumes that there is a linear relationship between the predictors and the log odds of the outcome. This property can lead to oversimplification of the models created to manage complex health data. Similarly, the research by Dufera et al. (2023) focuses on the application of linear regression (LR) to quantify the estimation of obesity rates in various communities. The system is built on the basic assumption of integration between obesity rates and other socio-economic factors such as income, education, and even exercise amenities. The process entailed estimation of the linear regression model, which sought to find a straight line that minimized the sum of the squared errors between the observed obesity rate and the predicted one. Nonetheless, due to its simplicity and straightforward interpretability, the study revealed that the application of LR encounters serious limitations, meaning that it is not capable of correctly dealing with many predictor relationships that form complex and non-linear patterns. This constraint decreases the model's reliability and feasibility in a wide variety of practical public health scenarios. Another study was conducted by Han et al. (2023), where the author aimed at comparing the discrimination ability of LogR and DTs in terms of mortality in infectious disease outcomes. It estimates the likelihood of a surge in relation to environmental and demographic data, while DTs were used to categorize the areas into risk zones. The authors also discovered that, as compared to DTRs, DTs gave further predictions by addressing non-linear associations and interaction results, though they considered problems such as overfitting, for instance, in studies with small sample sizes.

### 2.2 Machine learning approaches

Thanks to the development of machine learning (ML) technology, better and more effective predictions can be made about the health of the community. Real-life cardiovascular disease risk assessment was conducted in a study that used decision trees (DTs) to predict risk (Katsura et al., 2024). DT is used to divide the dataset so that the subset aligns with major health issues such as blood pressure, cholesterol levels, and smoking status. It works by selecting the best features at each node and minimizing impurities as measured by metrics such as the Gini index or entropy (Li, 2023). Another study related to community health assessment was presented, which investigated the use of support vector machines (SVMs) in predicting cancer outcomes. SVM works by finding the optimal hyperplane to maximize the gap between the outcomes of different classes, such as remission or relapse. The scheme works by converting the data into a higher-dimensional space using a kernel function that enables the SVM to handle non-linear relationships between features. Although SVMs achieve high accuracy, the complexity of the model and the difficulty of interpreting the results make them less suitable for public health applications. Similarly, the article (Alshanbari et al., 2023) analyzes the application of neural networks (NNs) in predicting mental health disorders and uses a multi-layered neural network in which each neuron is equipped with a weighted sum of inputs, followed by a non-linear activation function. It uses backpropagation techniques in which the network is iteratively adjusted with weights to minimize prediction errors. These scenarios capture complex patterns in the data but require large, computationally intensive datasets, which limits their application to public health.

### 2.3 Advanced ensemble methods

Sinha Roy et al. (2023) focused on the application of ensemble learning techniques and used random forests (RFs) in predicting chronic diseases. It constructs multiple decision trees based on random samples of data and then averages their decisions to produce the output. RFs consist of a bootstrap aggregating (bagging) process, where each tree is built using a distinct random sample with replacement. This method shrinks the coefficient estimates toward zero and thus reduces the variance and improves the model stability. The study also demonstrates that RFs are more accurate than single decision trees, especially when dealing with large datasets. Similarly, a study that has been discussed by Kitson et al. (2023) argued for the application of Bayesian networks (BNs) as a tool for assessing public health implications. This process entails the development of a probabilistic graphical model that captures the conditional dependencies of the health indicators. The BNs calculate the probability of the joint distribution of the variables and apply the probability of Bayes' theorem to compute the change in an outcome with evidence. The method allows for the natural representation of uncertainties through probabilities but involves structural learning that relies significantly on domain knowledge. This type of learning has been described in similar studies (Silva et al., 2023; Ordovas et al., 2023; Salve et al., 2023).

The problems associated with the techniques that have been discussed in this section, as far as public health predictions are concerned, have flaws in non-linearity, class labels, and over-fitting, and they cannot be generalized to new data. In response to the abovementioned problems, the proposed study employs an RFA approach. This helps avoid the problem of overfitting by building several decision trees on a bootstrap sample and then averaging their outputs. It also enhances generalization to new, unknown data and the adequacy to capture non-linear ties and multi-class expectations by considering many predictors and their interactions. Further, RFA predicts the probability of features by determining how much the performance of the model reduces if feature values are permutated.

## 3 Methodology

**Figure 1:**
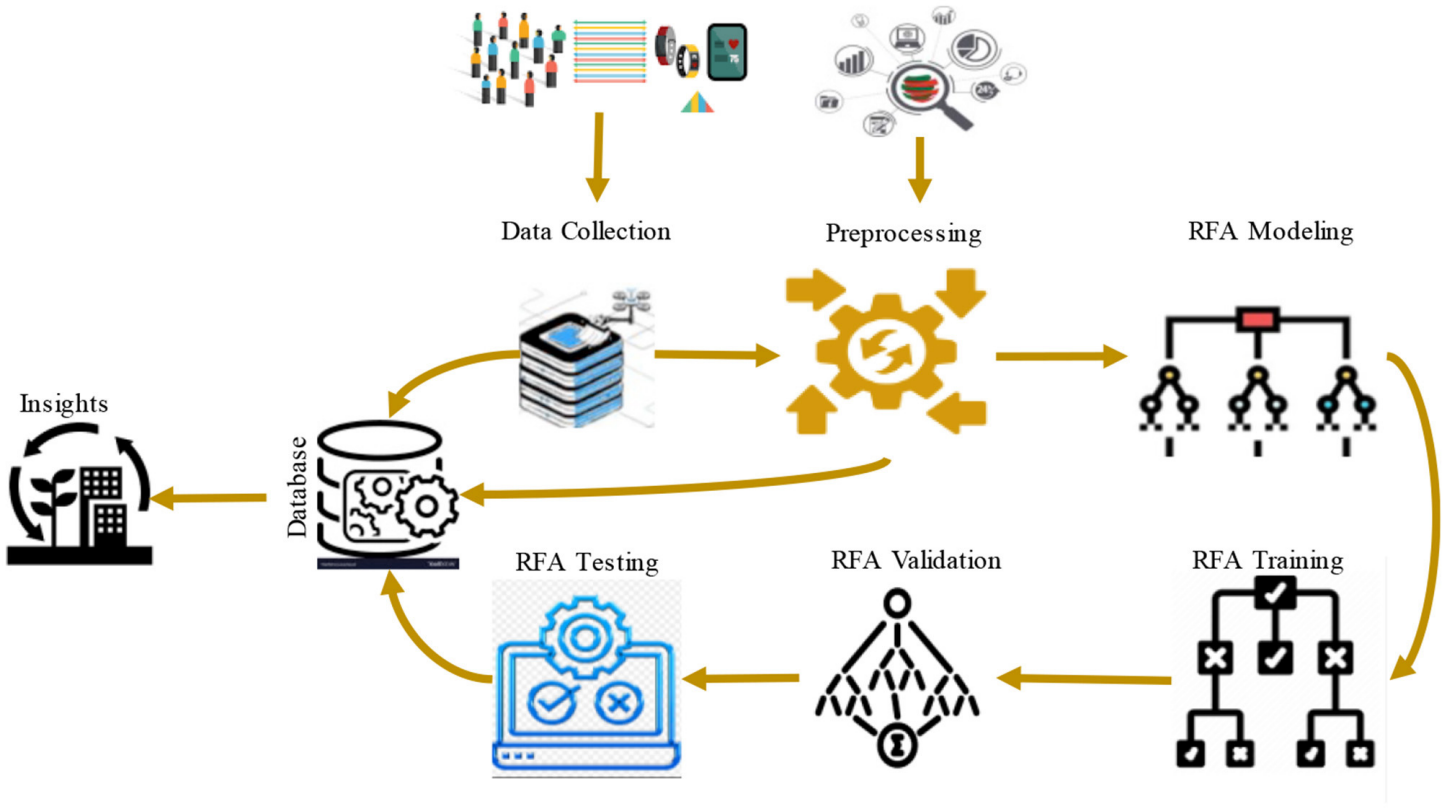
Process flow of the proposed RFA model.

In this section, an emphasis is placed on how the RFA can be used for the prediction of community health status. It provides a description of the data preprocessing techniques, model building, and assessment. It begins with data collection on various aspects of community health. Some of these records may include records concerning the public health and demographics of the area in question. The information is then preprocessed to eliminate mistakes and outliers, after which an extensive RFA model is developed. The dataset is then trained on the model, and the same is used to classify the data so that it predicts different conditions of the community health depending on the demographics. Figure 1 shows the process flow of the proposed RFA model.

### 3.1 Data collection

To make the proposed forecast more accurate and precise, this study obtains a wide variety of data to increase the accuracy and precision of the proposed forecast. This includes health metrics, demographic details, and socio-economic factors. Health metrics include blood pressure, body mass index (BMI), cholesterol levels, and sugar levels. All these levels provide a snapshot of individual health status. Second, there were demographic factors that included age, sex, ethnicity, and marital status. It provides information on how the state of health differs from one segment of the population to another. The third factor is socio-economic factors. Housing conditions, employment status, education, and income level are factors that compose a measure of the population and are used to examine the effect of socio-economic factors on health. Each of these attributes is of a different type, and all aspects of community health are investigated to examine them from different perspectives. The list of major attributes used in this study is described in Table 1.

**Table 1:** Process flow of the proposed RFA model.

**Attribute**	**Description**	**Type**
Blood pressure	Measurement of blood pressure levels	Health metric
BMI	Body mass index	Health metric
Cholesterol levels	Measurement of cholesterol levels	Health metric
Blood sugar levels	Measurement of blood sugar levels	Health metric
Age	Age of the individual	Demographic
Gender	Gender of the individual	Demographic
Ethnicity	Ethnic background of the individual	Demographic
Marital status	Marital status of the individual	Demographic
Income level	Annual income of the individual	Socio-economic
Education level	Highest level of education achieved	Socio-economic
Employment status	Current employment status	Socio-economic
Housing conditions	Quality of housing conditions	Socio-economic

The data is collected from a range of sources, which consist of health surveys, public health records, and socio-economic databases. Health surveys are conducted by local health departments and provide detailed health metrics and demographic information. Public health records provide information about historical data on health indicators, disease prevalence, and community health trends. Similarly, socio-economic data is obtained from national census databases and employment records. This multi-source approach ensures that the dataset is comprehensive, reliable, and represents the community's health landscape in an effective way. The chosen parameters are selected because they directly impact health outcomes.

Approximately 1,000 individual records were collected for this study. This large dataset includes detailed information on each of the major parameters and is essential for training the RFA effectively. The volume of the dataset with which the model is trained enables it to learn complex patterns and interactions between variables to make accurate predictions. Table 2 shows the distribution of the data in the dataset.

**Table 2:** Distribution of data in the dataset.

**Parameter**	**Percentage of total**
Blood pressure	100%
BMI	100%
Cholesterol levels	100%
Blood sugar levels	100%
Age	100%
Gender	100%
Ethnicity	100%
Marital status	100%
Income level	100%
Education level	100%
Employment status	100%
Housing conditions	100%

This detailed data collection process ensures that RFA can provide accurate and actionable insights into community health outcomes, which can support effective public health interventions and strategies. Table 3 shows the key health metrics used in this study. The mean values provide a general sense of the population's health, whereas the standard deviations indicate variability within the dataset. It also captures the range of the minimum and maximum values and ensures that the dataset includes both normal and extreme health conditions.

**Table 3:** Distribution of health data in the dataset.

**Health metric**	**Mean value**	**Standard deviation**	**Min value**	**Max value**
Blood pressure (mmHg)	120/80	130/85	90/60	180/120
BMI	25.4	4.5	18	35
Cholesterol (mg/dL)	200	30	150	300
Blood sugar (mg/dL)	90	20	70	140

The demographic information gathered from the participants is presented in Table 4. These factors include age, sex, and ethnicity. The age attribute is distributed in such a way that it shows a well-balanced representation of different age groups with a slight emphasis on middle-aged individuals. The attribute related to Gender is evenly distributed, while the ethnicity breakdown reflects the diversity in the community. These demographic variables are essential for understanding how health outcomes vary across different groups.

**Table 4:** Distribution of demographic data in the dataset.

**Demographic attribute**	**Category**	**Number of records**	**Percentage of total**
Age	18–24	5,000	10%
	25–34	10,000	20%
	35–44	12,000	24%
	45–54	8,000	16%
	55–64	7,000	14%
	65+	8,000	16%
Gender	Male	25,000	50%
	Female	25,000	50%
Ethnicity	Caucasian	20,000	40%
	African American	15,000	30%
	Hispanic	10,000	20%
	Other	5,000	10%

Another key aspect of the dataset is the socio-economic indicators. Table 5 outlines the socio-economic data collected in the study, including income level, education, employment status, and housing conditions. The data indicates that many participants fall within the middle-income and higher education categories. This socio-economic information helps in analyzing the social determinants of health and their effects on the community's wellbeing.

**Table 5:** Distribution of socio-economic data in the dataset.

**Socio-economic factor**	**Category**	**Number of records**	**Percentage of total**
Income level	< $25,000	12,000	24%
	$25,000–$50,000	18,000	36%
	$50,000–$75,000	10,000	20%
	>$75,000	10,000	20%
Education level	High School	15,000	30%
	Some College	10,000	20%
	Bachelor's Degree	15,000	30%
	Master's Degree	7,000	14%
Employment status	Employed	35,000	70%
	Unemployed	10,000	20%
	Retired	5,000	10%
Housing conditions	Good	30,000	60%
	Fair	15,000	30%
	Poor	5,000	10%

**Figure 2:**
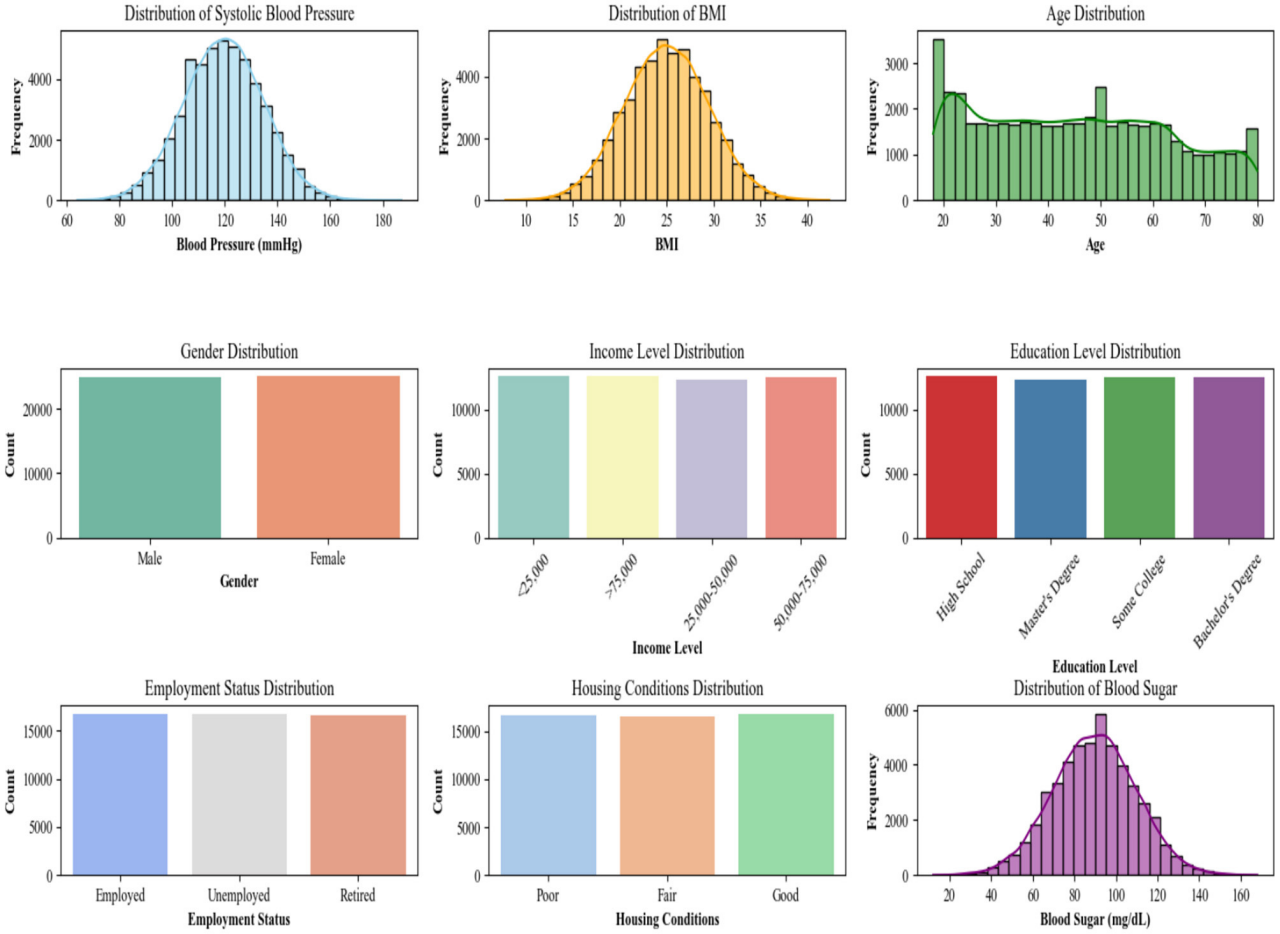
Distribution of the attributes in the dataset used for Community health prediction.

The variables of the dataset are distributed differently in the dataset. Each attribute has a specific number of values and ranges. Figure 2 shows the distribution of attributes in the dataset.

**Figure 3:**
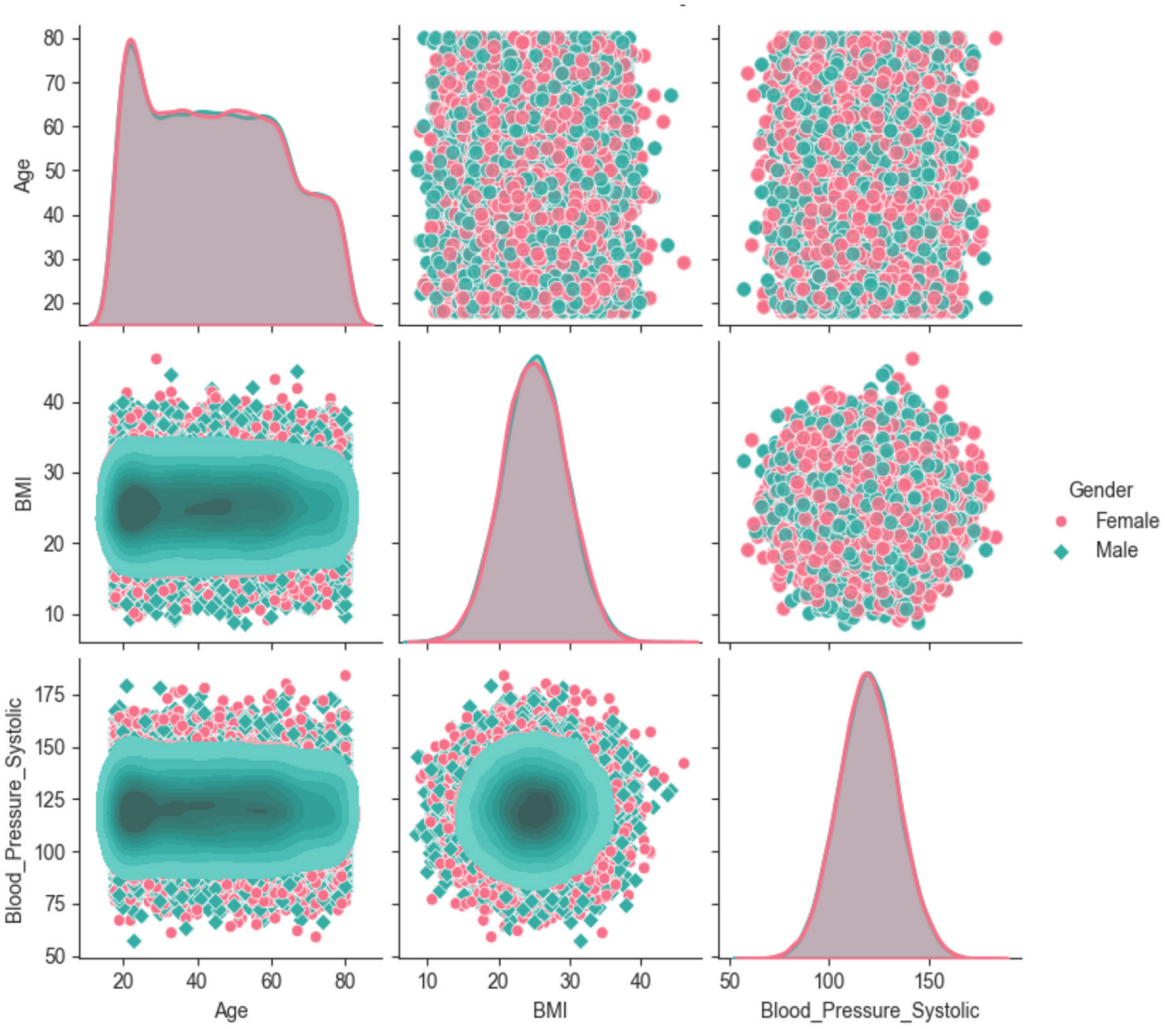
Correlation among different attributes of the dataset.

Each attribute of the dataset has a relationship with other attributes of the dataset. Some are directly dependent, and some are indirectly dependent on each other. Understanding the interactions between variables is helpful in identifying patterns that affect overall health outcomes. Key variables, such as Age, BMI, Blood Pressure, and Income, tend to interact in complex ways, which reveal potential correlations and associations. For example, an increase in age often correlates with important risk factors for chronic diseases, such as higher BMI and blood pressure. Similarly, lower-income groups typically experience higher health risks due to limited access to healthcare and healthy living conditions. Figure 3 shows the interactions among variables and assists policymakers in observing the strength and direction of the correlations.

**Figure 4:**
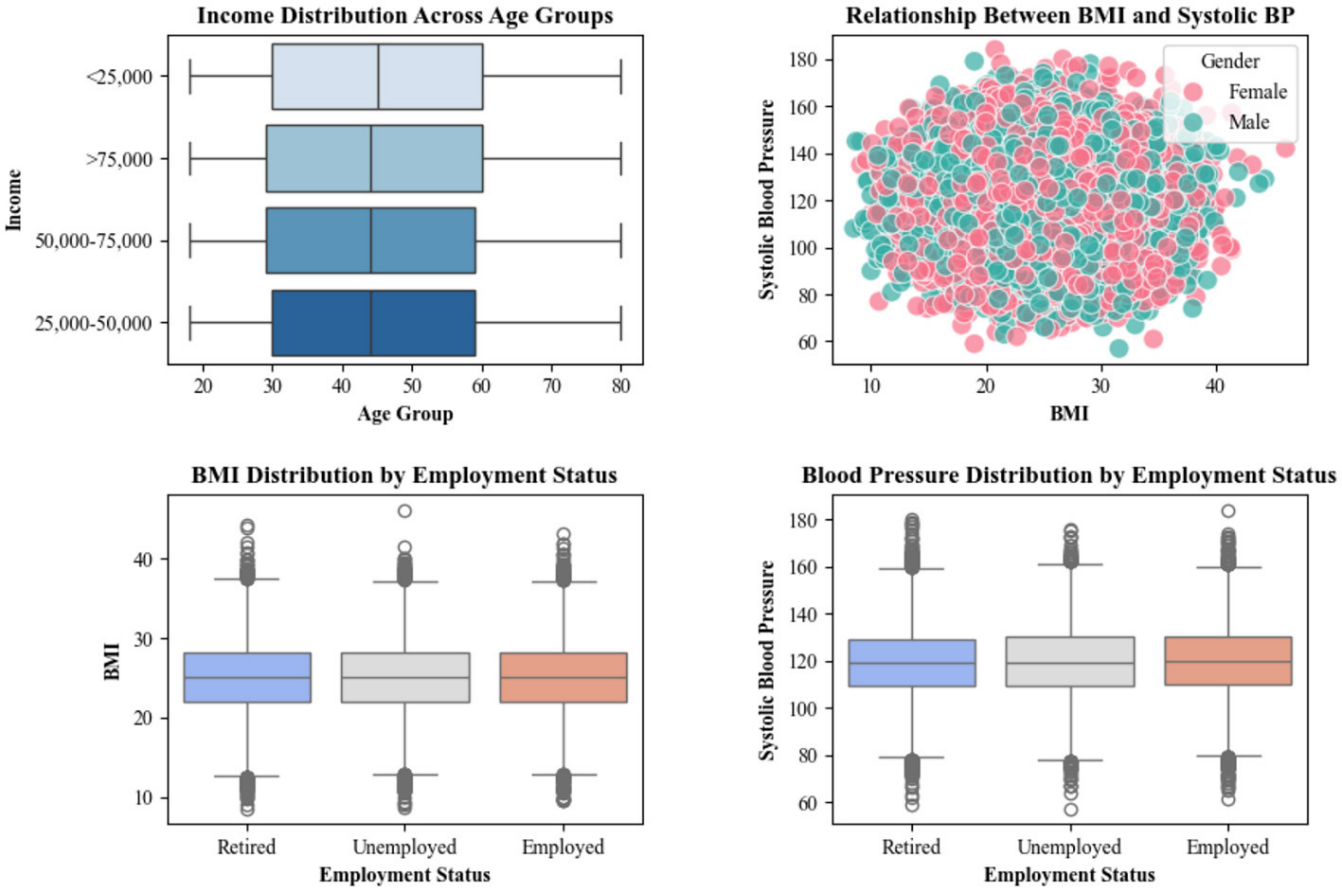
Impact of variables on affording health services.

The dataset trends show that income tends to vary significantly across different age groups in such a way that middle-aged individuals generally earn more, which is a sign that age plays a crucial role in income disparity and has a tendency to affect access to healthcare. Additionally, a clear relationship exists between BMI and systolic blood pressure, which indicates that individuals with higher BMI are more likely to experience elevated blood pressure. Finally, employment status also impacts health. As unemployed individuals tend to have higher BMI and blood pressure, this shows the influence of socio-economic factors on overall health. Figure 4 shows the Impact of variables on affording health services.

### 3.2 Data preprocessing

This phase of the study involves cleaning and preparing the dataset for the analysis. The collected data may not be complete for all attributes. It contains missing values, outliers, anomalies, and normalized and uniform data. This study focuses on key steps such as handling missing values, data normalization, categorical encoding, outlier detection, and feature selection. To remove the anomalies, different techniques are used. The first is the handling of missing values. The value of an attribute is not always available, or it may not be properly filled. This causes the attribute value to be missing. To deal with missing data, one common approach is mean imputation (Bhushan et al., 2024). The equation for mean imputation is


(1) $$\begin{array}{l} {\text{x}}_{\text{i}}=\frac{\overset{\text{n}}{\underset{\text{j}=1}{\sum }}{\text{x}}_{\text{j}}}{\text{n}} \end{array}$$

Where *x*_*i*_ is the missing value for a given feature, *x*_*j*_ is the observed value for the same feature in other records, and *n* is the number of non-missing values for that feature. In the dataset, there are missing data for the ‘BMI' column in 3 records. The mean of all available “BMI” values is computed, and the missing values are replaced with that mean. Suppose the available values are 20, 22, 24, and 26. The imputed value would be


(2) $$\begin{array}{l} x_{i}=\frac{20\;+\;22\;+\;24\;+\;26}{4}=23 \end{array}$$

After filling in the missing data from different records. The data should be adjusted in a uniform and normalized manner. To achieve this, Data Normalization is used. In this scheme, data is scaled to a fixed range, most probably between 0 and 1. The technique used for normalization is Min–Max scaling (Raza et al., 2024), which is mathematically represented by


(3) $$\begin{array}{l} x_{norm}=\frac{x-x_{\min}}{x_{\max}-x_{\min}} \end{array}$$

Where *x*_*norm*_ is the normalized value, *x* is the original data point, *x*_*min*_ is the minimum value in the dataset, and *x*_*max*_ is the maximum value in the dataset. In the utilized dataset, the income column ranges from $20,000 to $80,000. For a particular data point where *x* = 50,000, the normalized value would be


(4) $$\begin{array}{l} x_{norm}=\frac{50,000-20,000}{80,000-20,000}=\frac{30,000}{60,000}=0.5 \end{array}$$

Third, one of the most important features of the dataset is the encoding of the categorical variables. These ML models use vector data as input and do not recognize or process categorical data. ML models work best with numerical data. Some features may be in the form of texts or categories. These must be encoded before analysis. To make the dataset fit for ML models, RFA, in this case, encoding should be performed. This study uses a one-hot encoding approach. This involves converting categories into binary vectors. Suppose that the gender variable has two categories, Male and Female. By using one-hot encoding, it becomes


(5) $$Gender=\{\begin{array}{cc} (1,0), & \;if\;Male\\ (0,1), & \;If\;Female \end{array}$$

If the Gender values are like [Male, Female, Male], it is encoded as


(6) $$\begin{array}{l} Gender=\left[\left(1,0\right),\left(0,1\right),\left(1,0\right)\right] \end{array}$$

One of the features to make the dataset clean is the detection and removal of outliers. Outliers in the data must be handled carefully, as they are data points that differ significantly from the rest of the data. These points can skew the analysis or affect the performance of the ML models. It can be detected using statistical methods such as the Z-score or Interquartile Range (IQR). Once identified, a decision must be made to remove or adjust the data. Some models are sensitive to outliers, while others are robust. The Z-score for each data point is calculated as


(7) $$\begin{array}{l} Z_{i}=\frac{x_{i}-\mu }{\sigma } \end{array}$$

Where Z_i_ is the Z-score for the data point and x_i_ is the value of the data point, μ is the mean of the data and σ is the standard deviation of the data given by Equation 8.


(8) $$\begin{array}{l} \sigma =\sqrt{\frac{1}{N-1}\overset{N}{\underset{i=1}{\sum }}{\left(x_{i}-\bar{x}\right)}^{2}} \end{array}$$

Where *x*_*i*_ sis the value of the dataset, $\bar{x}$ is the mean of the selected value, and *N* is the total number of values. The Blood Pressure column has a mean of 120 mmHg and a standard deviation of 15 mmHg. For a data point where *x*_*i*_ = 150, the Z-score would be


(9) $$\begin{array}{l} Z_{i}=\frac{150-120}{15}=2 \end{array}$$

As per the previous assumption, a Z-score value above 3 or below −3 is typically considered an outlier. Feature selection is another key component in this phase. The dataset contains many attributes. However, these attributes are not equally important. Some are more important, whereas others are less important. To decrease the overall processing power and increase the robustness of the model, only the most relevant features were selected. This also reduces the issue of overfitting. Feature selection techniques help in identifying the most important features, and methods such as correlation analysis, variance thresholding, and recursive feature elimination are commonly used. This step helps in reducing complexity and improving model performance. The correlation shows the relationship between two variables, and it is computed as


(10) $$\begin{array}{l} r_{xy}=\frac{\sum \left(x_{i}-\bar{x}\right)\left(y_{i}-\bar{y}\right)}{\sqrt{\sum {\left(x_{i}-\bar{x}\right)}^{2}\sum {\left(y_{i}-\bar{y}\right)}^{2}}} \end{array}$$

where r_xy_ is the correlation between variables *x* and *y*; x_i_ and y_i_ are the individual data points of *x* and *y*, respectively; and $\bar{x}$ and $\bar{y}$ are the means of *x* and *y*, respectively. In the proposed dataset, by considering income values and


(11) $$\begin{array}{l} \;Age=\left[30,40,50,60,70\right],\\ Income=\left[40,000,50,000,60,000,70,000,80,000\right] \end{array}$$

**Figure 5:**
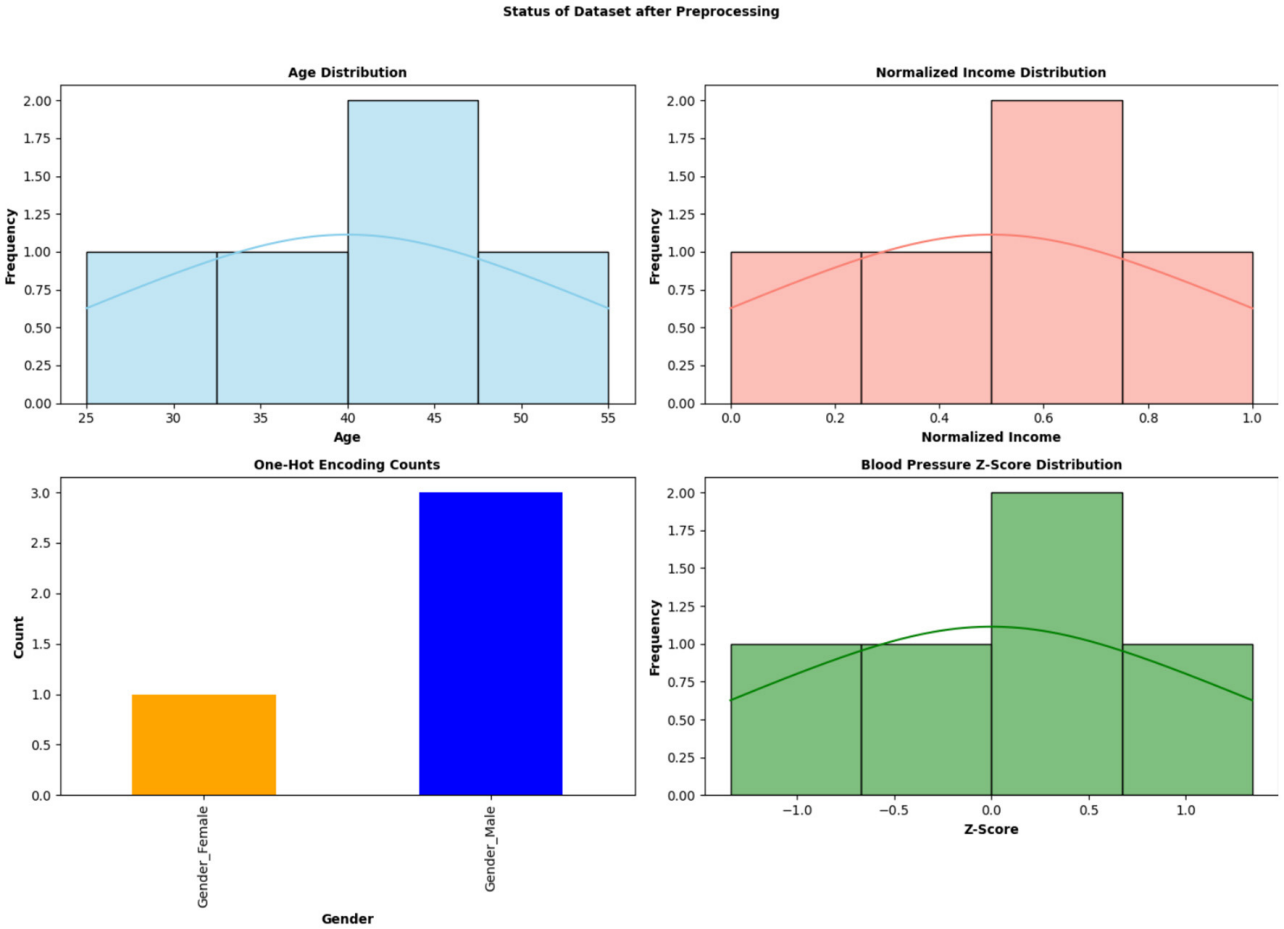
Analysis of the dataset using preprocessing techniques.

The correlation between “Age” and “Income” can be computed using the above formula. A result close to 1 indicates a strong positive correlation. In the end, data transformation techniques, such as log transformation and polynomial transformation, are applied to the dataset. These schemes help in improving the relationships between features. Log is useful when there are exponential relationships in the data, while a Polynomial helps in capturing non-linear relationships. Data transformation can lead to improved model accuracy. Figure 5 shows the preprocessing of the dataset.

### 3.3 Random forest algorithm

The dataset of community health is collected and then preprocessed for the removal of noise, unwanted anomalies, and errors. It also transforms the dataset into a specified format such that it can be utilized by an ML Algorithm. The proposed study uses RFA, which is an ensemble learning method that combines the predictions of multiple decision trees to achieve superior performance. The algorithm is divided into several major steps, which include data sampling, tree construction, feature selection, aggregation of predictions, and feature importance evaluation. Each of these steps has a pivotal impact on the performance of RFA. These techniques are described below.

#### 3.3.1 Data sampling

The first step of RFA is to divide the dataset and create multiple subsets of the original dataset. This is achieved through a process known as bootstrapping. Each subset of data, also known as a bootstrap sample, is used to train an individual decision tree. This process ensures that each tree is trained on a slightly different dataset so that the overall generalization of the model is improved and the variance of the model operationally is reduced. To create a bootstrap sample, the algorithm randomly selects data points from the original dataset *D* with replacement. This process continues until the bootstrap sample has the same number of samples as that of the original dataset. For instance, if the original dataset contains patient records with attributes such as age, blood pressure, and cholesterol levels, each bootstrap sample will contain a random selection of these records. The result of bootstrap sampling creates a new dataset $D_{i}^{*}$ for each decision tree *T*_*i*_. Ley says *D* represents the original dataset with *n* samples. The bootstrap sample $D_{i}^{*}$ is generated by random sampling with replacement. For each tree *T*_*i*_, the bootstrap sample $D_{i}^{*}$ is created as follows:


(12) $$\begin{array}{l} D_{i}^{*}=\left\{\left(x_{j1},y_{j1}\right),\left(x_{j2},y_{j2}\right),\ldots ,\left(x_{jn},y_{jn}\right)\right\} \end{array}$$

where (x_ji_, y_ji_) are randomly selected with replacement from *D*. Each bootstrap sample most probably includes approximately 63% of the original samples in such a way that some samples appear multiple times and others do not appear at all. This is also used to reduce overfitting. For the utilized health dataset with 1,000 patient records, a total of 100 bootstrap samples are generated, each containing 1,000 records. Each of the 100 decision trees is trained on a different bootstrap sample, which constructs a number of trees; all trees then combine to create an RF. Table 6 provides sampling details for the study.

**Table 6:** Bootstrap sampling details.

**Sample number**	**Data sample size**	**Percentage of total data**
Sample 1	70% of the dataset	70%
Sample 2	70% of the dataset	70%
Sample 3	70% of the dataset	70%
…	…	…
Sample 100	70% of the dataset	70%

#### 3.3.2 Tree construction

The second step of the proposed algorithm is to construct individual decision trees using bootstrap samples. These trees are built using recursion, in which the data are divided based on the selected features until a stopping criterion is met. The goal is to create trees that capture different aspects of the data. Each decision tree is constructed using respective bootstrap samples. The process works in such a way that at each node of a tree, a subset of features is randomly selected, which determines the best split. This randomness helps reduce the correlation between trees and enhances model diversity. The techniques used for splitting the dataset are based on Gini impurity or mean squared error (MSE). These are used to find the optimal feature and split point. For a node *n* in a decision tree, a subset *F*_*i*_ of features is chosen from the total set *F*. The Gini impurity for classification is described mathematically as


(13) $$Gini(n)=1-\;\sum_{c=1}^{C}p{\left(c|n\right)}^{2}$$

where *p(c| n)* is the probability of class *c* at node *n*. For regression, the mean squared error is calculated as follows:


(14) $$MSE\left(n\right)=\frac{1}{\left|T_{n}\right|}\sum_{\left(x,y\right)\in T_{n}}{\left(y-y\right)}^{2}$$

where y^−^ is the mean target value at node *n*. Each decision tree length is dependent on the maximum depth or termination criteria already defined. The trees are not pruned and retain as much information as possible. The number of decision trees is 100, with a maximum depth of 15. A depth of 15 was selected to balance the model complexity and interpretability. Each tree uses a random subset of features at each node to determine the splits based on Gini impurity. Algorithm 1 describes the process of tree construction by splitting the dataset into multiple trees.

**Algorithm 1:**
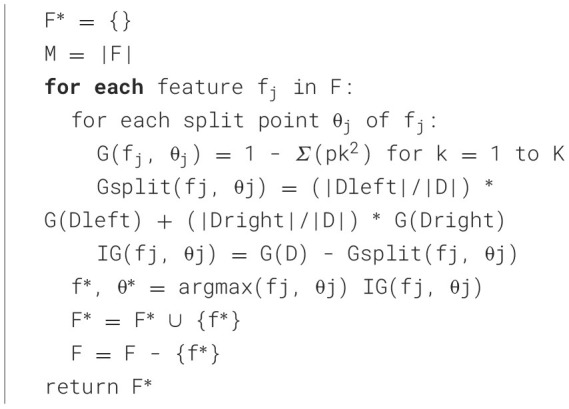
Classification of nodes in multiple trees.

The feature-selection algorithm for RFA (Algorithm 2) works by iterating each feature and determining the optimal split point based on the Gini Index. This index is used to measure the purity of the data in such a way that, in each feature and split, the algorithm calculates the Information Gain, which depicts the improvement in classification. A feature that maximizes gain is selected. This process is repeated for all features and split points, and the best features are added to the final set. Table 7 describes the tree constructions used in this study.

**Algorithm 2:**
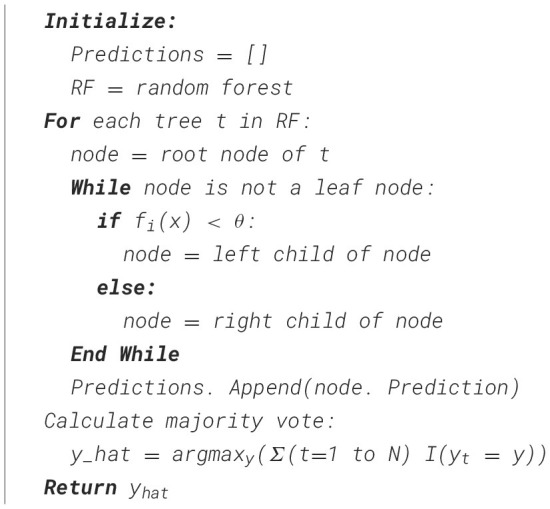
Prediction of health using RFA.

**Table 7:** Random forest hyperparameters.

**Hyperparameter**	**Value**	**Description**
Number of trees	100	Number of trees in the Random Forest
Max features per split	3	Number of features randomly selected per split
Bootstrap sample size	70%	Percentage of data used for each bootstrap sample
Minimum samples per leaf	5	Minimum number of samples required to be at a leaf node
Maximum tree depth	15	Depth of the trees (trees grow until all leaves are pure)

#### 3.3.3 Feature selection

This feature of the study involves the selection of a subset of features such that each feature is considered a node of the decision trees. It introduces randomness and helps in reducing the correlation between trees, which improves model performance and stability. A random number of features is selected from the dataset, namely *F*. Each node of the tree represents the feature as *F*_*i*_. where *I* is the number of features. The size of this subset is determined based on whether the task is classification or regression. For classification tasks, subset size is typically calculated using the following equation:


(15) $$\begin{array}{l} F\;=|\sqrt{\left|F\right|} \end{array}$$

The best feature f* for splitting is selected based on the following equation:


(16) $$\begin{array}{l} f^{*}=\arg Max_{f\in F_{i}}Criterion\left(f\right)\; \end{array}$$

where *Criterion(f)* is the Gini impurity used in the proposed study. It is used to introduce randomness into the tree-building process and helps in the reduction of correlation between trees and improving the overall model performance. By taking only a subset of features at each node, the proposed RF model captures a broader range of patterns. In this dataset, within a total of 20 features, RFA selects a random subset of 4 features at each node for the classification tasks. The feature selection phase should ensure that the features are not overlooked and that the model remains effective by capturing different aspects of the data. Table 8 describes different features selected for this study.

**Table 8:** Features selected from the dataset used in this study.

**Feature**	**Importance score**	**Selected for model**
Blood pressure	High	Yes
Income	High	Yes
Chronic disease status	Medium	Yes
Age	Medium	Yes
Education level	Low	Yes
Housing condition	Low	No
Employment status	Low	No
Gender	Low	No

Importance Score reflects how much each feature contributes to reducing impurity and improving model accuracy, and the attribute Selected for Model indicates whether the feature is included in the final RF model based on its importance score.

#### 3.3.4 Aggregation of prediction

This phase is related to the combination of outputs of all decision trees in the RF, which produces the final prediction. Each decision tree provides a prediction for a given sample in such a manner that the final prediction is obtained by aggregating these individual predictions.


(17) $$\hat{y}=mode\left(\left\{T_{1}\left(x\right),T_{2}\left(x\right),\ldots ,T_{m}\left(x\right)\right\}\right)$$

where *T*_*i*_*(x)* is the prediction of the tree T_i_ for sample *x*. The aggregation helps in reducing the variance of the model and improving overall accuracy. The challenge in this process is the decision that each tree contributes meaningfully to the final prediction. If 70 out of 100 decision trees predict class A and 30 predict class B, the final prediction will be class A. Table 9 presents different trees constructed with tree resampling.

**Table 9:** Feature subsampling for tree construction.

**Tree number**	**Features considered**
Tree 1	Blood pressure, income, age
Tree 2	Chronic disease status, income, education level
Tree 3	Blood pressure, age, education level
Tree 100	Income, chronic disease status, blood pressure

#### 3.3.5 Feature importance evaluation

**Figure 6:**
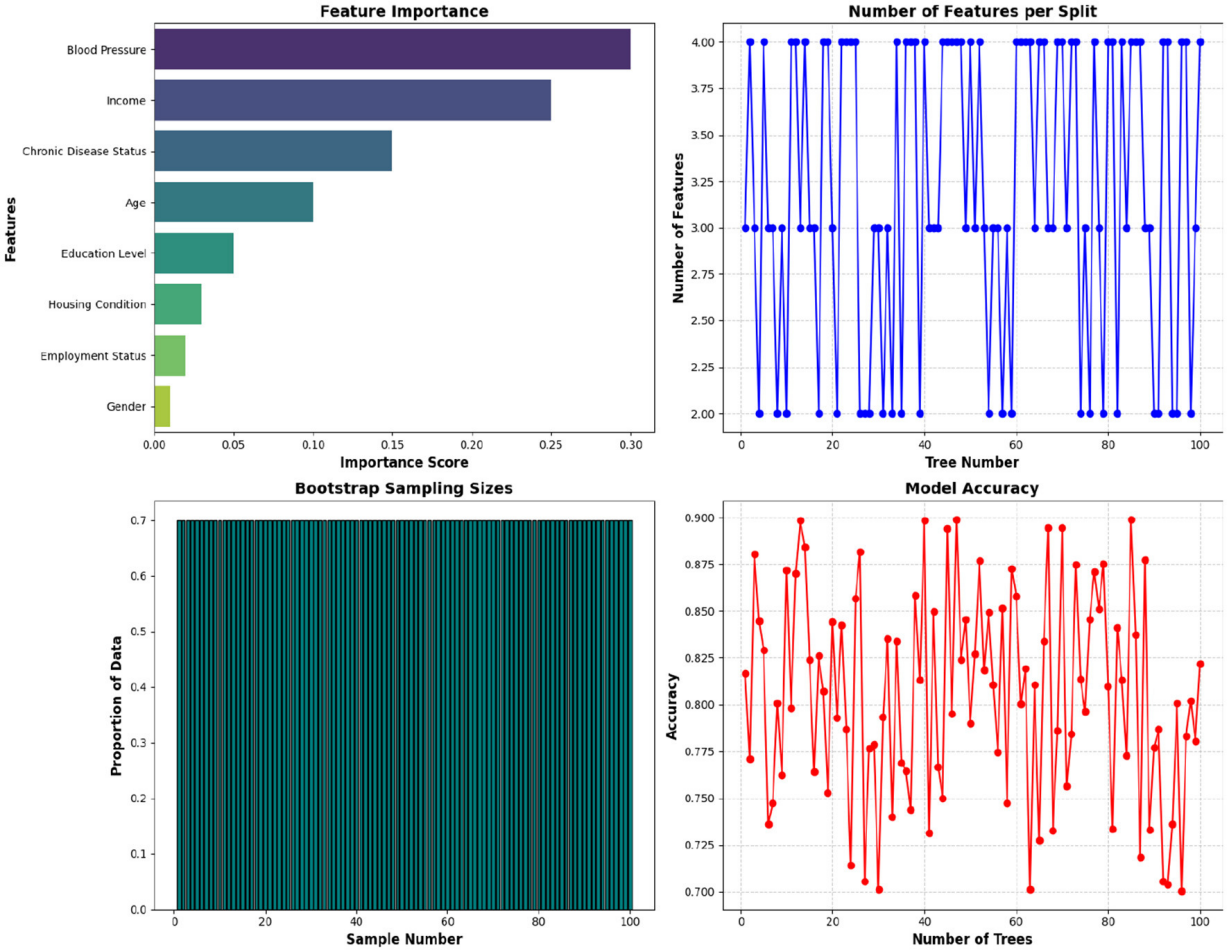
Properties of the proposed RFA for Community Health.

Feature importance evaluation involves assessing the contribution of each feature to the model's predictions. This step helps in identifying which features are most influential in predicting health outcomes and can be useful for feature selection and interpretation. It is calculated by measuring the extent to which each feature decreases the impurity of nodes across all trees and reflects the feature's contribution to improving the model's predictions. The step helps in identifying which features are most influential in predicting health outcomes and can be useful for feature selection and interpretation. Figure 6 shows various properties of the proposed model.

The algorithm for the prediction of community health conditions based on data is depicted below.

**Figure 7:**
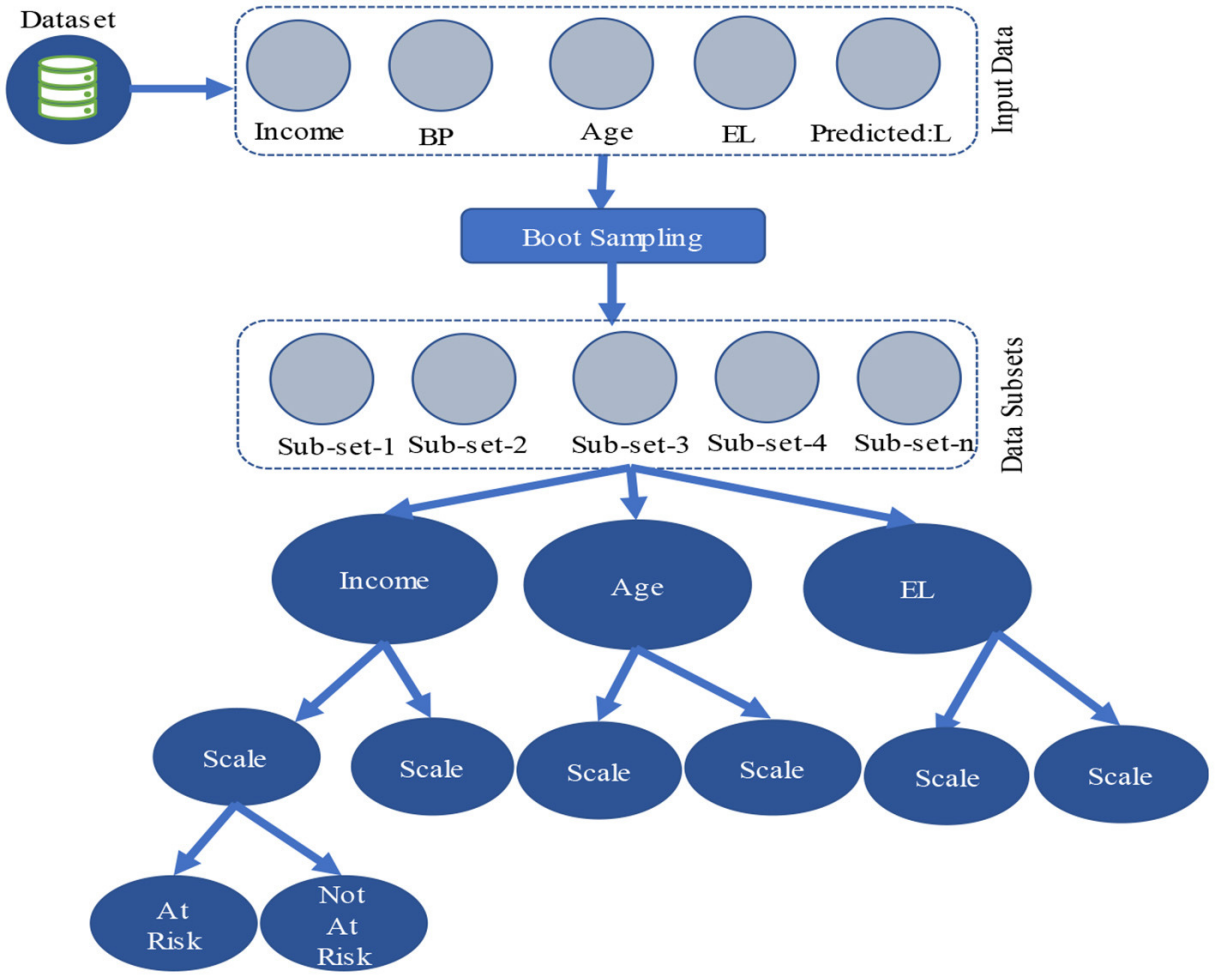
The Proposed RFA for the Community Health Predictions.

Let us consider whether there is a prediction that a patient has a high risk of developing a chronic disease, based on their blood pressure, income, age, and education level. Suppose the input data point comprises different variables with data as blood pressure of 140 mmHg, income of $50,000, age of 55 years, and education level of high school. Each tree in the Random Forest evaluates this input and produces a prediction of either ‘High Risk' or “Low Risk.” If 80 out of 100 trees predict High Risk, the Random Forest aggregates these predictions and classifies the patient as High Risk. This approach reduces the likelihood of overfitting and improves the model's overall predictive accuracy. Figure 7 shows the RF for the proposed study with the prediction of Low Risk and High Risks.

RFA is a powerful and flexible method for forecasting and classification using available data. It creates multiple decision trees with different subsets of data and features and aggregates its predictions for achieving high accuracy and robustness.

### 3.4 Model training

After the model is constructed, it should be trained, validated, and then tested on different data subsets. Hyperparameters, such as the number of trees, the maximum depth of each tree, and the number of features to consider at each split, are tuned to optimize the model performance. After the model is trained, the model is cross-validated, which evaluates the model during training and avoids overfitting. The training process iteratively adjusts the model parameters, which improves prediction accuracy. Once the model is trained, it is evaluated using a separate test dataset.

## 4 Results and discussion

After successful training and testing, the model needs to be evaluated using different metrics and parameters. It assesses how well the RFA performs in predicting outcomes related to community health. The evaluation is ascertained by simulating the RFA on collected datasets and comparing its predictions against actual values. The process helps ensure the model's accuracy, precision, and overall reliability. It is also helpful for analyzing errors, testing generalizability, and checking for overfitting. To overcome this issue, the cross-validation technique is used, which splits the data into training and testing sets, allowing the model to be tested multiple times. The simulation scenario focuses on community health prediction based on socio-economic and health parameters such as age, blood pressure, income, housing conditions, and employment status. The model was simulated in a controlled environment with varying levels of missing data, training sizes, and trees in the forest. Each simulation replicates a real-world scenario in which incomplete or noisy data are often encountered in health applications. The simulation environment is depicted in Table 10.

**Table 10:** Simulation environment of the proposed model.

**Component**	**Description**	**Details**
Simulation platform	The platform used for running the simulations	Jupyter Notebook
Operating system	The OS used for running the simulation	Windows 10
Programming language	The language used for coding the simulation	Python 3.10
Execution time	Time taken for the simulation to run	~15 min
Random seed	Seed to ensure the reproducibility of results	Fixed Seed (e.g., 42)
Development IDE	Integrated Development Environment (IDE)	Jupyter Notebook
Software libraries	Libraries used in the simulation	Scikit-learn, pandas, seaborn, matplotlib
Cross-validation	Number of folds used in cross-validation	10-fold cross-validation

The RFA model designed in the proposed study describes the architecture of processes and methodologies used to validate and evaluate the proposed RFA model. The model is designed to optimize the performance and accuracy of predictions in the community health study. It consisted of 100 decision trees such that each tree was constructed using a bootstrapped sample of the data. This method allows for the creation of multiple diverse trees that collectively improve overall prediction accuracy. The maximum depth for each tree is limited to 15 meters, which is used to ensure a balance between complexity and efficiency. This will avoid overfitting while capturing the underlying patterns of the data. The simulation parameters used in this study are depicted in Table 11.

**Table 11:** RFA model environment.

**Parameter**	**Description**	**Value/method**
Number of trees	Total number of decision trees in the Random Forest	100
Max depth	Maximum depth of each decision tree	15
Bootstrap sampling	Random samples with replacement to create different training subsets	Enabled
Split criteria	Criterion to evaluate the split points in each tree	Gini Index
Feature subset size	Number of features randomly selected for node splitting in each tree	√(Number of Features)
Training data size	Proportion of the dataset used for training the model	80% of the dataset
Testing data size	Proportion of the dataset used for testing the model	20% of the dataset
Cross-validation	Number of folds used in cross-validation to evaluate model performance	10-fold cross-validation
Random seed	Seed to ensure the reproducibility of results	Fixed Seed (e.g., 42)

Different performance metrics are used to evaluate the performance. The performance metrics are described as follows. The first and most important factor is the determination of the number of correct and incorrect predictions. These values were gauged using four main techniques: true positive, true negative, false positive, and false negative. True positives are values that occur when the predicted label matches the actual label model and correctly identifies an instance. Conversely, a True Negative occurs when the model nullifies the condition, i.e., both the predicted and actual labels are negative. On the other hand, a False Positive, also known as a Type I error, arises when the model incorrectly predicts the positivity of an instance and generates a false alarm. Finally, a False Negative (Type II error) occurred when the model failed to detect a condition that was present. By testing the proposed model, different values of these performance metrics are measured, which are depicted in Table 12.

**Table 12:** Total number of instances identified as TP, TN, FP, and FN.

**Metric**	**Value**
True Positives (TP)	480
True Negatives (TN)	450
False Positives (FP)	30
False Negatives (FN)	40
Total Records	1,000

**Figure 8:**
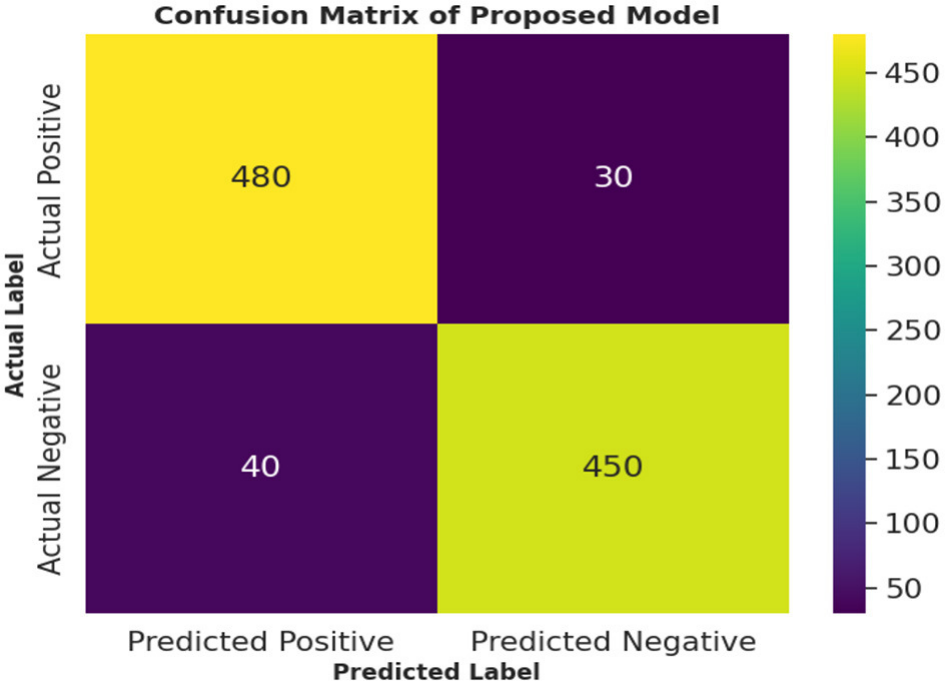
Confusion matrix of the proposed model.

A total of 1,000 records are analyzed using the proposed RFA for the community health study. The details showed that the model yielded 480 true positives and 450 true negatives, which indicates a high degree of accuracy in correctly classifying positive and negative outcomes. The false positives are 30 cases, and the false negatives are 40 cases, which are fewer in number and showcase the proposed method's ability to minimize misclassification. Figure 8 shows the confusion matrix for the proposed study.

Another important aspect of the performance of the proposed study is the Receiver Operating Characteristic (ROC). It graphically represents the classification performance of a model by plotting the True Positive Rate (TPR) (also called Sensitivity or Recall) against the False Positive Rate (FPR) at various threshold settings.


(18) $$\begin{array}{l} TPR=\;\frac{TP}{TP+\;FN}\;=\;\frac{480}{480\;+\;40}\;=\;0.923 \end{array}$$

(19) $$\begin{array}{l} FPR=\;\frac{FP}{FP+\;FN}\;=\;\frac{30}{30\;+\;450}\;=\;0.062 \end{array}$$

**Figure 9:**
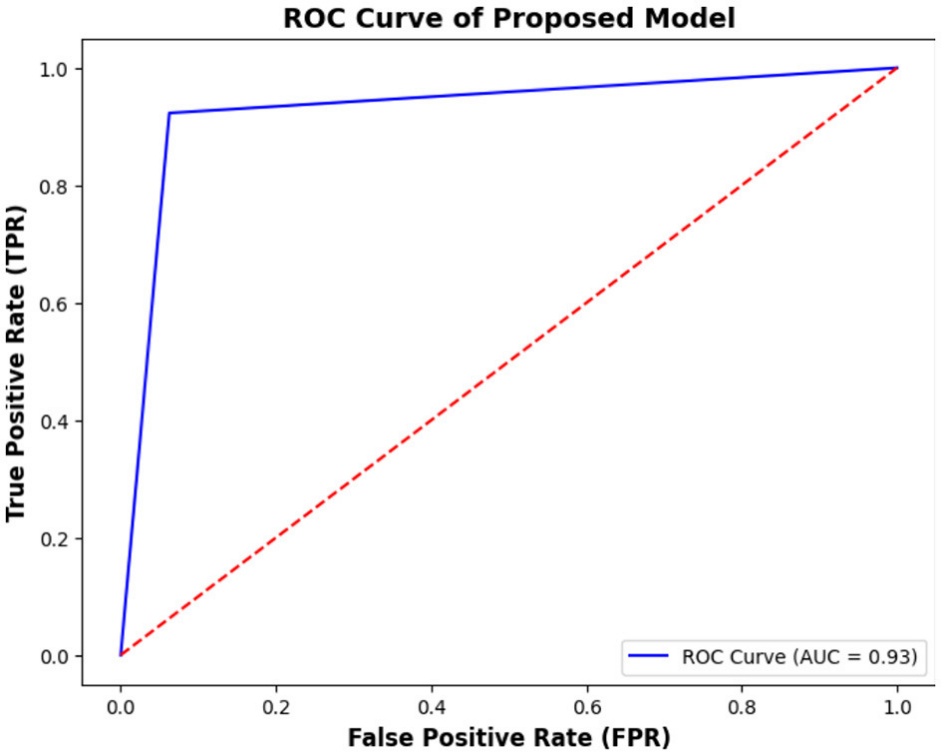
ROC curve for the proposed model.

Where TP is true positive, FP is false positive, and FN is false negative. The ROC curve displays the trade-off between TPR and FPR for different classification thresholds. A high TPR with a low FPR represents good performance in forecasting and vice versa. Figure 9 shows the ROC curves of this study.

To compare the performance of the proposed model with other state-of-the-art models, it is compared with a Decision Tree (DT), Support Vector Machine (SVM), and Neural Networks (NN). The metrics used to compare these models with the proposed model are Accuracy, Precision, Recall, F1-score, and log. The first and most important performance metric is Accuracy. The term accuracy refers to the degree of correctness achieved by a model. Mathematically, this is expressed using the following equation:


(20) $$\begin{array}{l} Accuracy\;=\frac{TP+TN}{TP+FP+TN+FN} \end{array}$$

**Figure 10:**
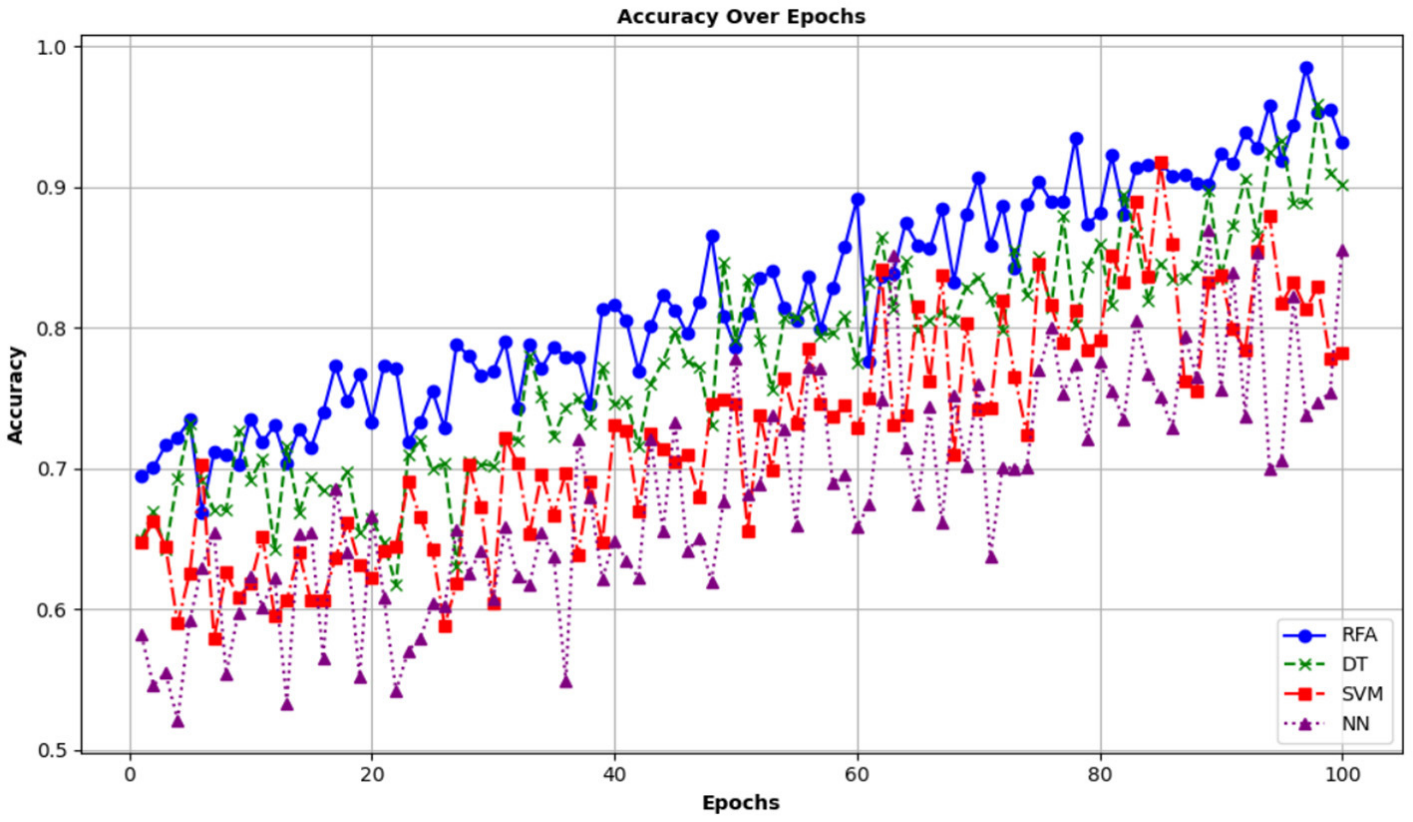
Accuracy of different ML Models in predicting community health.

Where *TP* denotes true positive values, *FP* shows False positives, *TN* is true negatives, and *FN* is False negatives. A high accuracy of a model depicts that the model has high capability in predicting true cases. It means that the RFA is effectively identifying the correct outcomes of community health, which include healthcare access and resource allocation. Figure 10 shows the accuracy metrics of the proposed study.

The second indicator that demonstrates the performance of the model is precision. Precision is defined as the percentage of true positive predictions in a set of positive predictions. It is calculated using the following equation:


(21) $$\begin{array}{l} Precision=\;\frac{TP}{\left(TP\;+\;FP\right)\;} \end{array}$$

**Figure 11:**
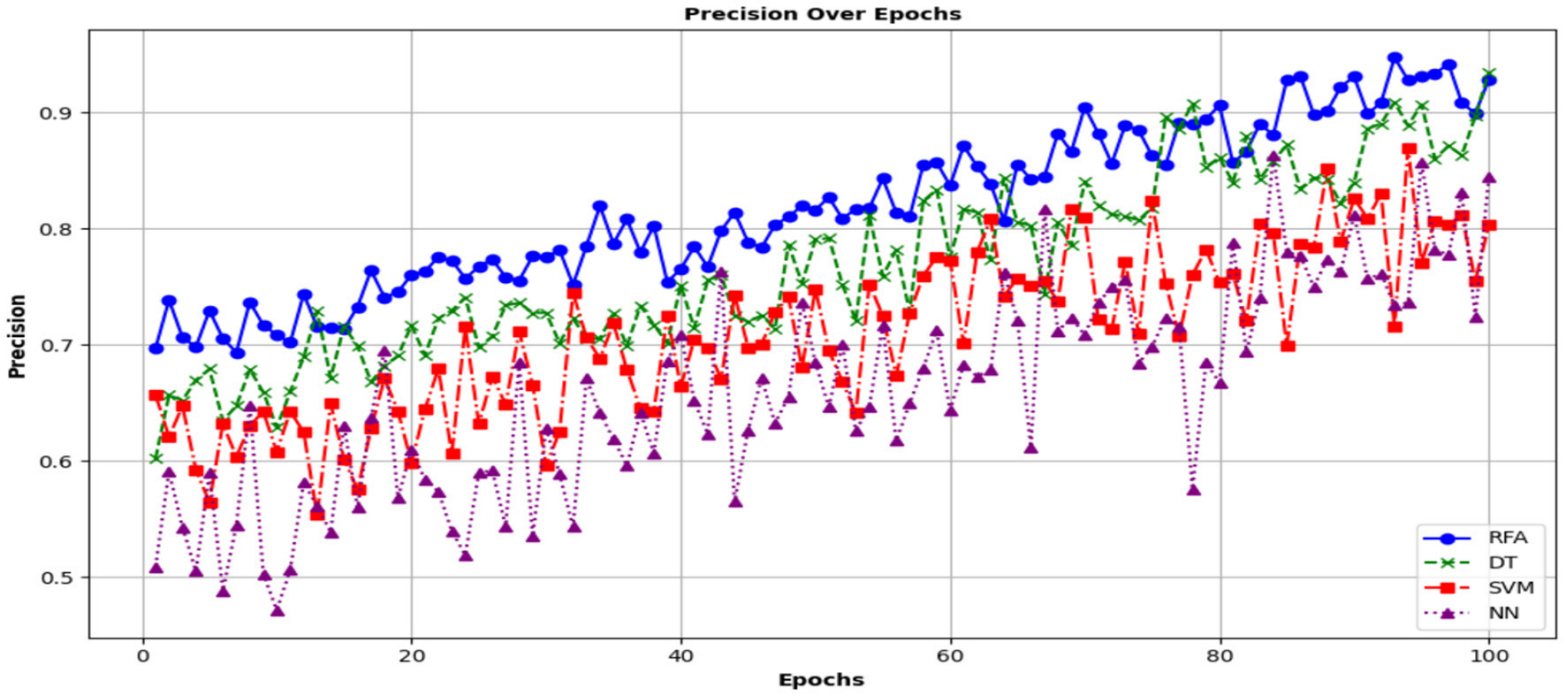
Analysis of community health in terms of precision.

A high value of precision depicts that when the model predicts a forecast, the model is most probably correct. It shows the model is performing better in identifying community health-related issues and persons who are infected. Figure 11 shows a precision analysis of the different models.

The trend of the graph shows that the model begins and shows improvement over time compared to the other models. This is also a sign that the proposed model is more effective in the accurate prediction of healthcare-related outcomes, with a precision of approximately 0.90 by the 20th epoch. The third performance metric is Recall, which is also called sensitivity. It is used to determine the capability of the models to define the correct cases. It is calculated mathematically as the ratio of true positives to the sum of true positives and false negatives, as shown in the equation below:


(22) $$\begin{array}{l} Recall=\;\frac{Tp\;}{TP+FN} \end{array}$$

**Figure 12:**
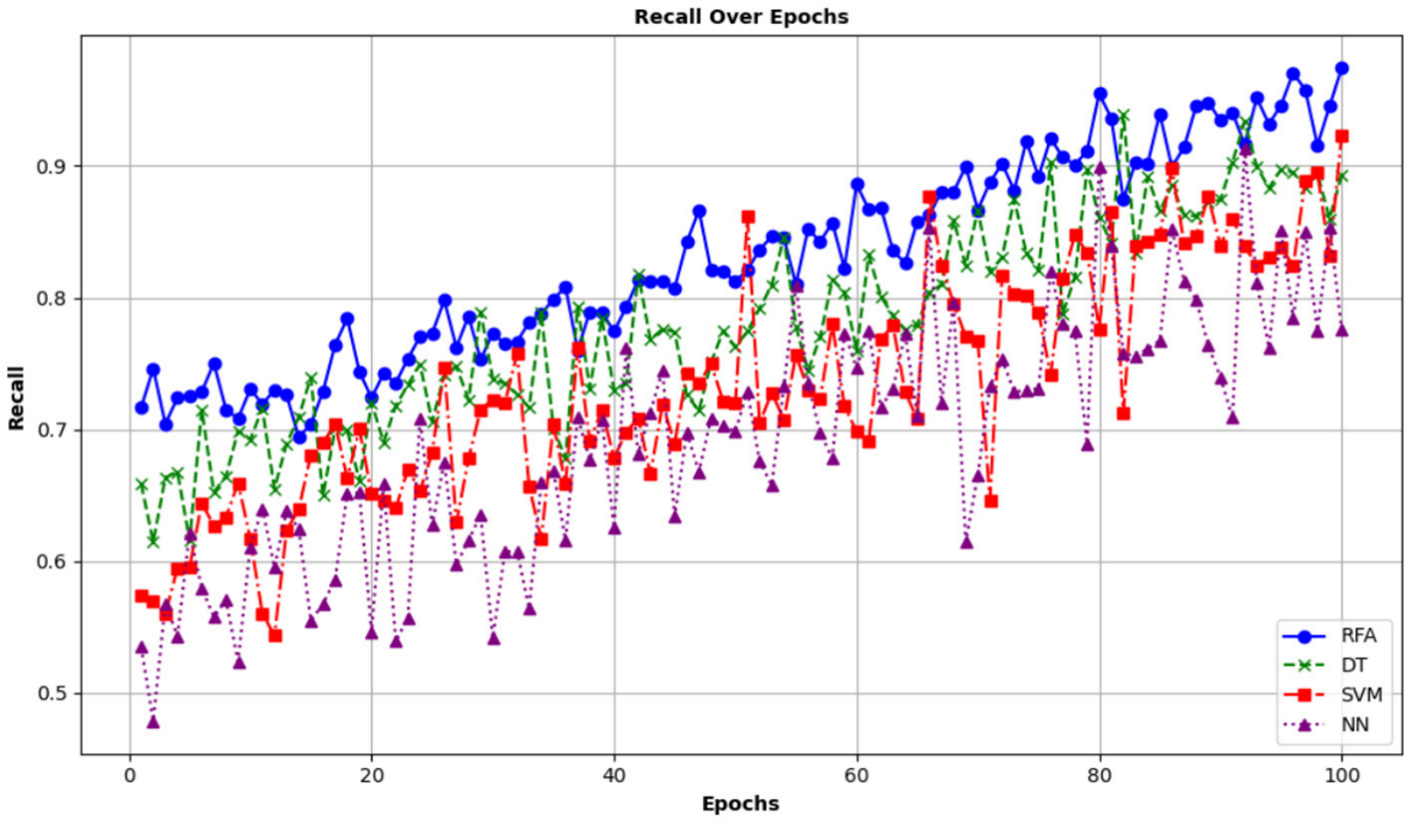
Comparison of recall among various prediction models.

A high degree of recall indicates that the model is successful in identifying the positive cases. This minimizes the chance of missing cases and confirms that most individuals with a disease are correctly identified. The recall value measured from the said study is compared with other models and is demonstrated in Figure 12.

The fourth performance metric is the use of the F1 score. This metric is used to define the balance that exists between precision and recall. Mathematically, this can be illustrated as follows.


(23) $$\begin{array}{l} F1\;Score=2\;X\;\frac{Prec\;X\;Recall}{Prec\;+\;Recall} \end{array}$$

**Figure 13:**
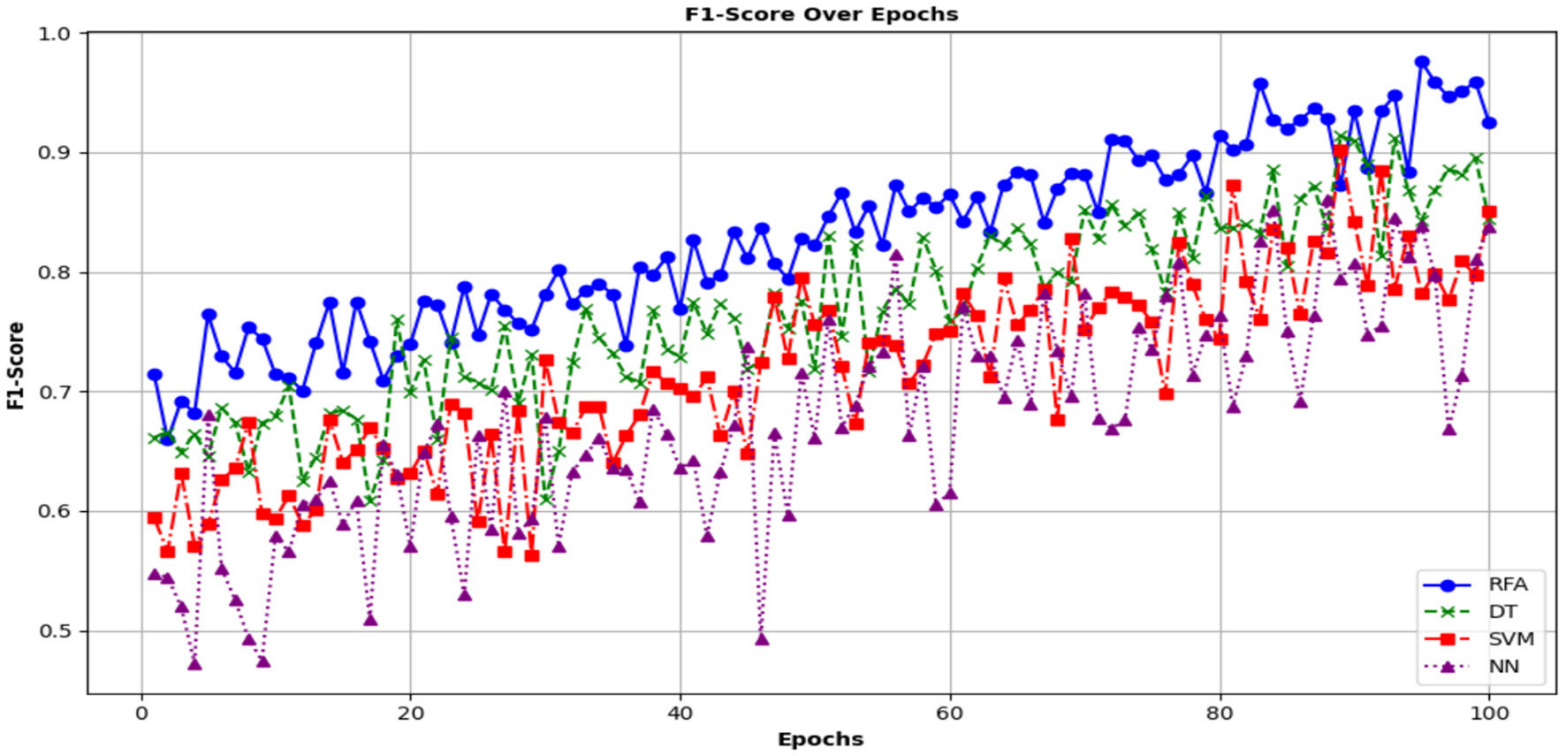
F1 score analysis in terms of different ML models.

This indicator is useful for balancing the trade-off between precision and recall. Figure 13 shows the F1-score computed for the accuracy and precision of the different models.

**Figure 14:**
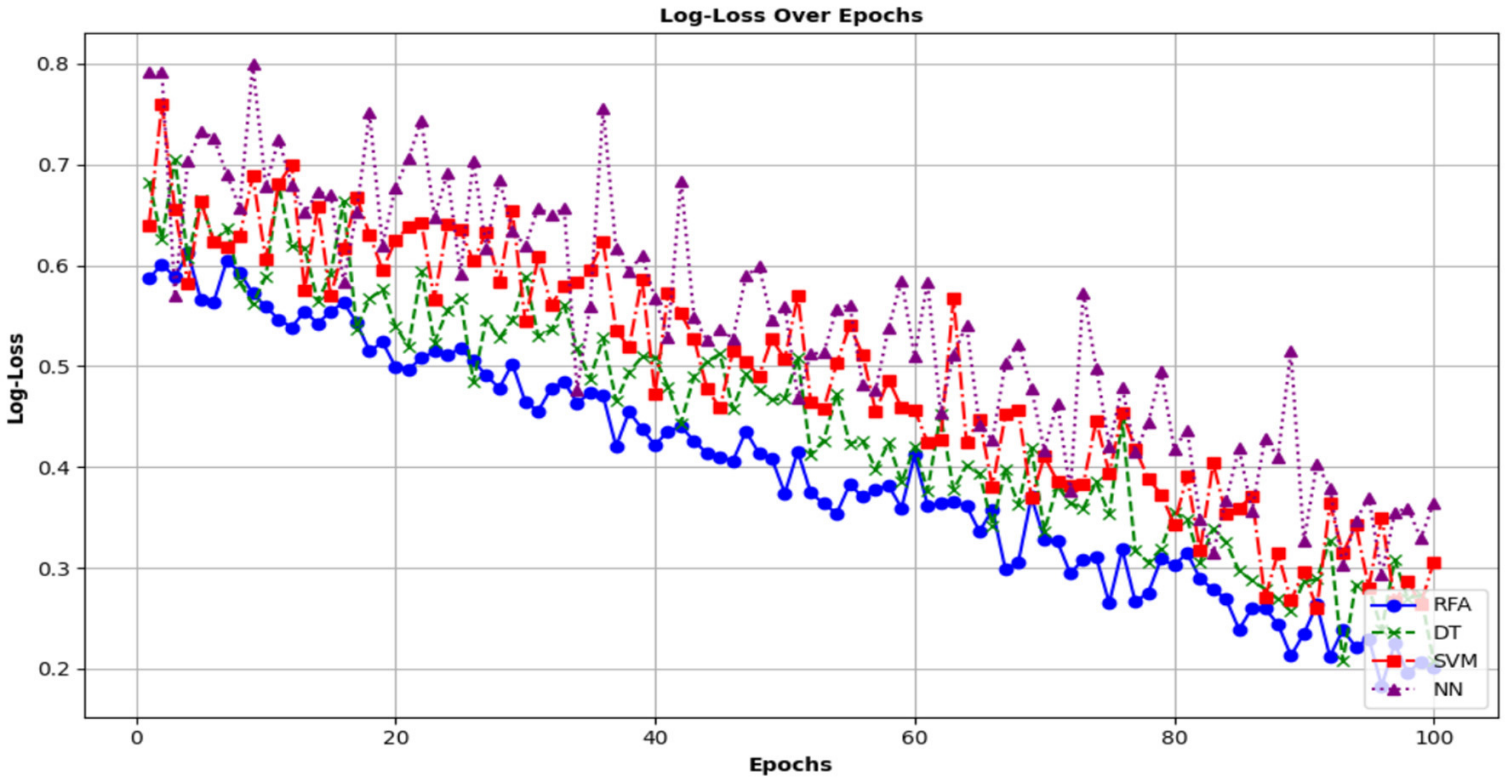
Analysis of Log-Loss metric for different models of community health.

The last performance metric is the Logarithmic Loss (Log-Loss) function. This is used to measure the performance of a model whose output is based on a probability value. It defines the aberration between the total predicted probabilities and the actual binary outcomes. Mathematically, it is calculated using the following equation:


(24) $$\begin{array}{l} Log-Loss=-\frac{1}{N}\;\overset{N}{\underset{i=1}{\sum }}\left[y_{i}\;\log\left(p_{i}\right)+\left(1-y_{i}\right)\log\left(1-p_{i}\right)\right] \end{array}$$

where *n* is the number of data points. *y*_*i*_ shows the actual label of the *i*^*th*^ data point in such a way that *y*_*i*_ ε{0,1} while *p*_*i*_ demonstrates the predicted probability of the *i*^*th*^ data point. This is due to more accurate probability predictions and vice versa. Figure 14 shows the log-loss function of the RFA model.

**Figure 15:**
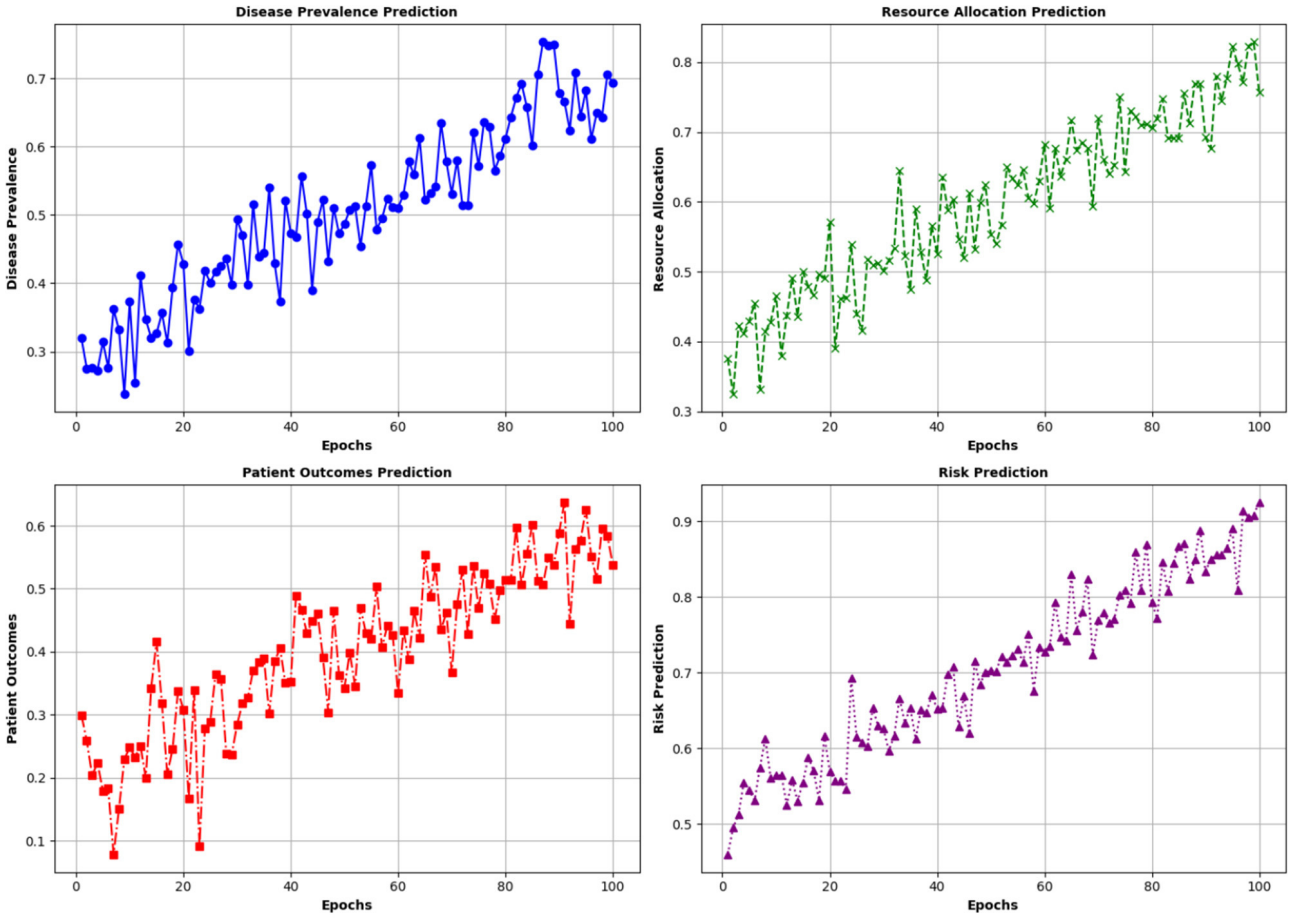
Effects of the model on different community health indicators.

The evaluation of the RFA model shows that it has a significant impact on the prediction of various factors of community health by constructing and defining thresholds for a collection of decision trees. The proposed model effectively analyzes and predicts key health indicators, which include disease prevalence, resource allocation, and patient outcomes. It can identify patterns and trends in health data, which leads to more accurate forecasts of disease outbreaks and better management of healthcare resources. This predictive capability is also important for implementing timely interventions and optimizing healthcare strategies. The results of the simulation study show that the number of epochs is directly proportional to the degree of correct predictions by the RFA Model. This is because model training enhances the model's capability to predict more accurate results in the forecasting conditions of community health. The improvement in the quality of the model affects the prediction capability, which results in better decision-making and community health outcomes. Figure 15 shows the impact of the RDA on community health.

## 5 Conclusion

This study investigated the impact of MLM, specifically RFA, on forecasting the conditions of community health by analyzing various health factors such as disease prevalence, resource allocation, patient outcomes, and risk prediction. This study collected data from sources related to community health. The collected data is then transformed into a uniform format and preprocessed by removing errors and anomalies. Subsequently, it was trained using a portion of the dataset. The training process is divided into different epochs. After training, the model is tested to forecast the status of community health. The performance of the proposed model is assessed by comparing it with other algorithms, such as Decision Trees (DT), Support Vector Machines (SVM), and Neural Networks (NN). The results show that the proposed MLM Model performs better than its predecessors in multiple metrics, which include accuracy, precision, recall, F1-score, and the log-loss function. This shows the high capability of RFA to handle complex interactions between features and its robustness against overfitting. This also shows the potential of ML techniques to make informed decisions in community health management and disease prevention. Future studies should focus on combining RFA with other ML techniques, such as deep learning models or ensemble methods, which could offer improved accuracy and robustness in the prediction of community health status.

## Data Availability

The original contributions presented in the study are included in the article/supplementary material, further inquiries can be directed to the corresponding author.
